# Organization and Integration of Care in the HIV–Non-Communicable Disease Syndemic: A Rapid Scoping Review

**DOI:** 10.3390/ijerph23050642

**Published:** 2026-05-12

**Authors:** Ketyllem Tayanne da Silva Costa, Maria Francisca da Conceição Maciel Targino, Pedro Ivo Torquato Ludugerio, Gidyenne Christine Bandeira Silva de Medeiros, Grasiela Piuvezam, Richardson Augusto Rosendo da Silva

**Affiliations:** 1 Department of Public Health, Federal University of Rio Grande do Norte, Natal 59078-970, Brazil; mfmaciel50@gmail.com (M.F.d.C.M.T.); pedro.torquato.076@ufrn.edu.br (P.I.T.L.); gidyenne.silva@ufrn.br (G.C.B.S.d.M.); grasiela.piuvezam@ufrn.br (G.P.); 2 Nursing Department, Federal University of Rio Grande do Norte, Natal 59070-405, Brazil; rirosendo@hotmail.com

**Keywords:** HIV, multimorbidity, chronic disease, integrated care, nursing

## Abstract

**Highlights:**

**Public health relevance—How does this work relate to a public health issue?**

- It addresses the HIV-NCD syndemic as a growing challenge that impacts morbidity and mortality and places a burden on health systems.
- It highlights gaps in integrated care and in the capacity of health services to meet the needs of people living with HIV and chronic conditions.

**Public health significance—Why is this work of significance to public health?**

- It examines how the integration of HIV-NCD services is being implemented and explores the perceptions of users and healthcare workers.
- It underscores the increasing importance of the HIV-NCD syndemic and the need to reorganize health systems toward integrated care.

**Public health implications—What are the key implications or messages for practitioners, policy makers and/or researchers in public health?**

- Integrated care models and nurse-led interventions improve HIV-NCD care, but control of non-communicable diseases remains limited.
- Addressing the syndemic requires strengthening health systems and developing context-adapted implementation strategies.

**Abstract:**

Advances in antiretroviral therapy have transformed infection with HIV into a manageable chronic disease, increasing the survival of people living with HIV, who are also undergoing a demographic aging process marked by the emergence of non-communicable chronic diseases. This study aims to map and analyze how the scientific literature addresses the organization and integration of care in the HIV-NCD syndemic, identifying implications for nursing and for health systems. This is a Rapid Scoping Review, using the databases PubMed, Scopus, CINAHL, and LILACS. Data synthesis was conducted using Microsoft Excel. The research was structured using the PCC framework: Population—people living with HIV (≥18 years); Concept—organization and integration of care in the HIV-NCD syndemic, including care models, care coordination, service integration, and the role of nursing; and Context—health services and systems. Twenty-three studies were included, most of which used qualitative methodology, were conducted in sub-Saharan Africa, and had predominantly female samples. This study demonstrated that the organization of care in the HIV-NCD syndemic remains predominantly characterized by fragmented models, which are insufficient to address the complexity of multimorbidity. Integrated care models emerge as a promising strategy; however, their effects remain limited in settings marked by health inequalities.

## 1. Introduction

In recent decades, advances in antiretroviral therapy have transformed infection with human immunodeficiency virus (HIV) from a fatal condition into a manageable chronic disease, significantly increasing the survival of people living with HIV (PLHIV). Consequently, this population has experienced a demographic aging process marked by the emergence of non-communicable chronic diseases (NCDs), which increases the complexity of the healthcare required. Recent epidemiological studies indicate that the prevalence of NCDs among PLHIV is high and often exceeds that observed in HIV-negative populations, thereby demanding a more comprehensive and integrated approach to care [1,2].

This scenario highlights the coexistence and interaction of multiple health conditions that mutually reinforce one another, not only in biologically synergistic terms but also within the context of social and structural determinants, thereby characterizing a syndemic pattern. The concept of a syndemic emphasizes that coexisting health conditions, such as HIV and NCDs, cannot be adequately understood or addressed in isolation, as they are shaped by factors such as social inequality, stigma, unequal access to healthcare services, and economic and cultural vulnerabilities [3].

The World Health Organization (WHO) and other institutions have advocated for the integration of NCD care into HIV programs, progressively shifting from vertical models of care toward integrated, person-centered approaches that address both HIV treatment and the management of chronic comorbidities across primary and specialized levels of care [1,4]. Despite these recommendations, health systems continue to face challenges related to service fragmentation, gaps in care coordination and continuity, as well as barriers associated with workforce training and service infrastructure [5].

In this context of complexity, nursing assumes a central role in the organization and coordination of care, particularly within integrated care models. Recent literature indicates that nurses perform functions ranging from health education and the promotion of self-care to the articulation across different levels of care, support for therapeutic adherence, and longitudinal patient monitoring. These roles are essential to ensure continuity and comprehensiveness of care in contexts of multimorbidity [6,7].

Although there is extensive literature on HIV and NCDs separately, the systematization of evidence on how health systems organize care in response to the syndemic remains limited, including how HIV-NCD integration occurs and what structural role nursing plays in this process. This study advances the field by articulating the organizational and structural dimensions of care, highlighting the strategic role of nursing in integration.

From this perspective, this study aims to map and analyze how the scientific literature addresses the organization and integration of care in the HIV-NCD syndemic, identifying implications for nursing and for health systems.

## 2. Materials and Methods

### 2.1. Study Design

This study constitutes a Rapid Scoping Review conducted in accordance with the methodology proposed by the Joanna Briggs Institute (JBI) for scoping reviews, as outlined in the JBI Manual for Evidence Synthesis. Scoping reviews are particularly appropriate when the objective is to map key concepts, types of evidence, and knowledge gaps within a given field, especially when the phenomenon under investigation is complex, heterogeneous, or emerging, as is the case of the HIV-NCD syndemic [8]. This study focused on the four major groups of NCDs, as classified by the WHO: cardiovascular diseases, cancer, diabetes mellitus, and chronic respiratory diseases [9].

A rapid approach was adopted due to the need for timely evidence synthesis, while maintaining methodological rigor and transparency in the procedures [10]. The concept of syndemics has been increasingly employed to understand the complexity of multimorbidity in contexts of social vulnerability and fragmented health systems [2,11,12]. Given that this concept encompasses biological, social, and structural dimensions, and that the aim of this study is to understand how care has been organized and integrated, a scoping review is methodologically appropriate.

### 2.2. Search Strategy

The research question was structured in accordance with JBI recommendations using the Population, Concept, and Context (PCC) framework: Population—people living with HIV (≥18 years); Concept—organization and integration of care in the HIV-NCD syndemic, including care models, care coordination, service integration, and the role of nursing; and Context—health services and systems. Accordingly, the guiding question of this study is: How has the scientific literature addressed the organization and integration of care in the HIV-NCD syndemic, and what are the implications for nursing and for health systems?

Based on this framework, the search strategy was developed following JBI methodological guidance for scoping reviews, using a structured three-step approach: (1) preliminary exploratory search, (2) development of the definitive strategy with controlled descriptors and free-text terms, and (3) adaptation to the selected databases [8].

An initial exploratory search was conducted in PubMed to identify indexed terms Medical Subject Headings (MeSH), recurrent keywords in titles and abstracts, and terminological variations related to multimorbidity and care integration. This step enabled the mapping of conceptual heterogeneity in the literature. Subsequently, the key terms identified were input into the GenAI tool ChatGPT, developed by OpenAI (version GPT-5.3 Instant, San Francisco, CA, USA), to identify descriptors most aligned with the study objectives and to support the development of the search strategy. The authors reviewed and edited the output and assumed full responsibility for the content of this publication.

It was observed that the term “syndemic” is still inconsistently used in empirical studies addressing HIV and NCDs. Therefore, the search was not restricted exclusively to the descriptor “syndemic,” in order to avoid loss of sensitivity. Instead, the syndemic concept was primarily incorporated during the interpretative stage of analysis. Descriptors related to nursing practice were also included in the search strategy, namely: “Nursing”, “Nursing Care”, and “Nurses” (MeSH terms), as well as “Nurse”, “Nursing Practice”, “Nursing Role”, and “Advanced Practice Nursing” (uncontrolled descriptors).

To ensure conceptual breadth, the main search focused on the organization and integration of care in the HIV-NCD syndemic. Given the analytical interest in nursing practice to better understand organizational processes and professional roles, a complementary search was conducted including specific descriptors related to nursing practice. The results were then combined and duplicates were removed using the Rayyan^®^ web platform prior to the screening stage.

**Figure 1:**
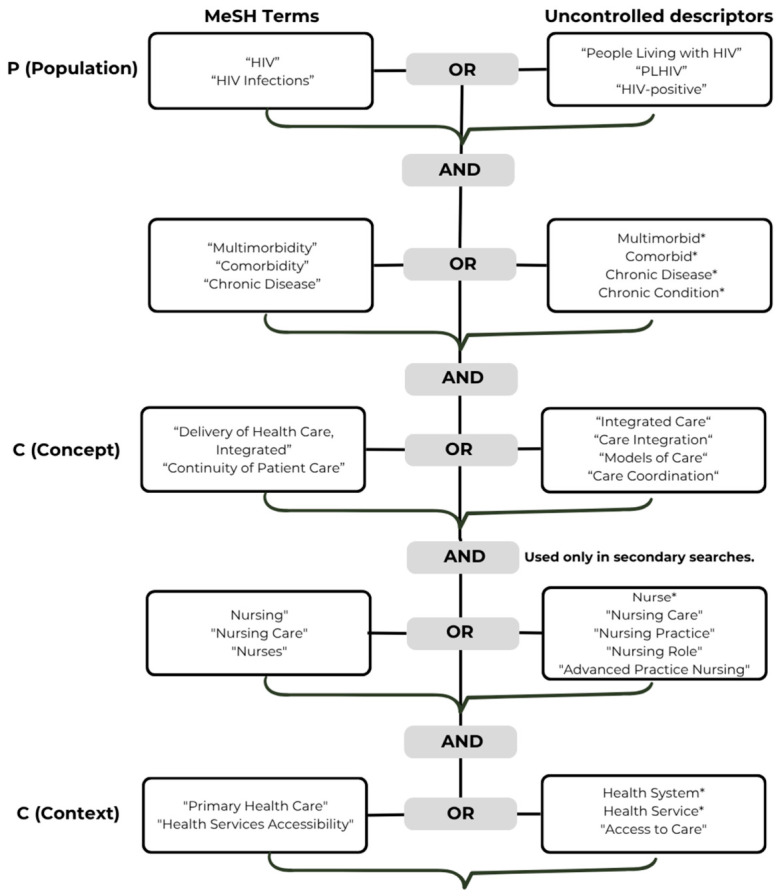
Structure of the search strategy according to the PCC framework (Population, Concept, Context). * Indicates truncation used to retrieve different word endings.

Figure 1 summarizes the search strategy employed, based on the combination of MeSH descriptors and free-text terms using Boolean operators (AND/OR), truncation (*), and quotation marks to delimit exact phrases, thereby ensuring a balance between sensitivity and specificity.

The search was conducted in the PubMed, Scopus, Cumulative Index to Nursing and Allied Health Literature (CINAHL), and Latin American and Caribbean Health Sciences Literature (LILACS) databases on 5 March 2026. Each search strategy was adapted to the specific indexing systems of the databases (MeSH, CINAHL Headings, and DeCS). The complete search strategies for all databases are provided in the Supplementary Materials (Table S1). Additionally, the search results were filtered by publication period (2010 to 2026) and language (English, Portuguese, and Spanish).

Studies published from 2010 onward were included, considering that this decade marks the consolidation in the literature of the recognition of multimorbidity among PLHIV, as well as an intensification of discussions on the reorganization of health systems and the integration of care in response to non-communicable chronic diseases [4,13].

### 2.3. Eligibility Criteria

Primary studies (quantitative, qualitative, or mixed-methods) were included, focusing on adult populations (≥18 years) with a diagnosis of HIV and at least one NCD. Eligible studies addressed at least one of the following topics: models of care organization, HIV-NCD service integration, care coordination, associated structural determinants, and the professional role of nursing. Publications between 2010 and 2026, in English, Portuguese, or Spanish, were considered.

Studies were excluded if they were exclusively biomedical or laboratory-based, pharmacological trials without an organizational perspective, pilot studies, pediatric studies, single case reports, or publications not subjected to peer review (e.g., editorials, letters to the editor, opinion papers, preprints), excluding also scoping reviews, systematic reviews, meta-analyses and meta-syntheses. Studies addressing only adherence to antiretroviral therapy (ART) without considering NCDs were also excluded. Veterinary studies were also excluded. Grey literature was not considered due to feasibility constraints inherent to the rapid scoping review methodology.

### 2.4. Screening

**Figure 2:**
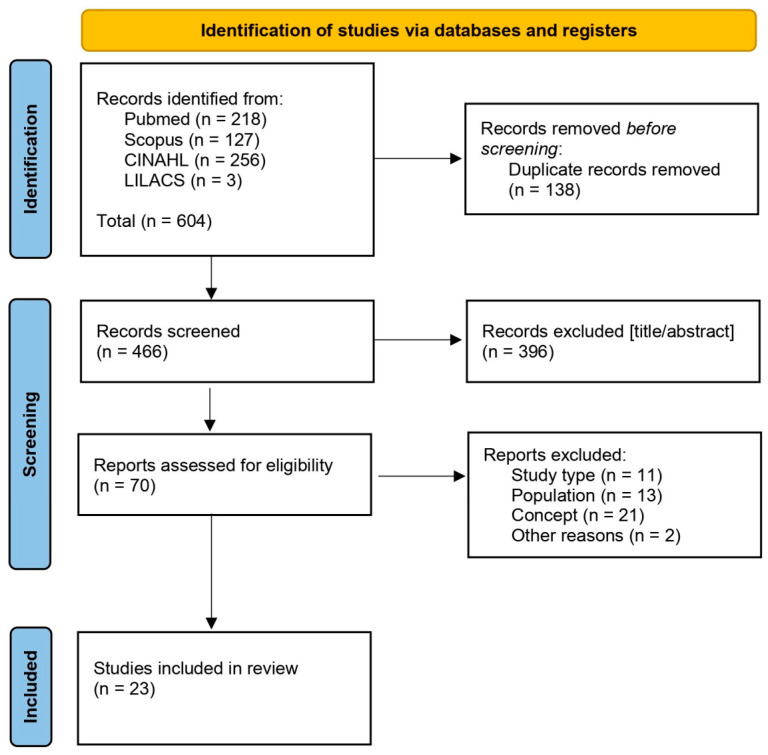
PRISMA 2020 flow diagram (modified for rapid scoping review) showing study identification, screening, eligibility, and inclusion.

The results were exported to the Rayyan^®^ software (Qatar Computing Research Institute, Doha, Qatar; version 1.7.3) for management and duplicate removal. The screening process was conducted in two stages: (1) title and abstract screening by two independent reviewers, and (2) full-text review of potentially eligible studies. Discrepancies were resolved by a third reviewer. The process is presented through a flow diagram in accordance with PRISMA-ScR (Preferred Reporting Items for Systematic Reviews and Meta-Analyses Extension for Scoping Reviews) [14], as shown in Figure 2.

### 2.5. Data Extraction

A standardized data extraction instrument developed by the authors was used, based on JBI recommendations. The extracted data included: author and year, country, study objective, methodological design, sample, sex, age profile, level of care (Table 1), NCDs investigated, care model and integration strategies, main challenges of the HIV-NCD syndemic, key findings (Table 2), the role of nursing, patients’ perceptions of nursing care, and the main implications for care (Table 3). These data are presented in the main text of this study. Additional extracted data can be found in the Supplementary Materials (Table S2).

### 2.6. Data Analysis and Synthesis

The data were analyzed using descriptive analysis and thematic analysis guided by the syndemic framework, supported by Microsoft Excel. Categorization was structured around three dimensions: (1) biological: HIV-NCD interaction, premature aging, and polypharmacy; (2) structural: social inequality, stigma, access to services, and social vulnerability; and (3) organizational: fragmentation or integration of care, care coordination, the role of primary health care (PHC), and the strategic role of nursing. The synthesis was narrative, with complementary tabular presentation. Reporting of the results followed the recommendations of the PRISMA-ScR checklist (File S1).

### 2.7. Methodological Quality Assessment

According to JBI guidance, scoping reviews do not require a mandatory critical appraisal of the methodological quality of included studies, as their primary aim is to map the available evidence rather than to assess effectiveness [8]. Therefore, no formal risk of bias assessment was conducted.

### 2.8. Ethical Aspects

As this is a secondary study using publicly available data, approval by a Research Ethics Committee was not required, in accordance with Resolution No. 510/2016 of the National Health Council [15].

## 3. Results

### 3.1. Study Selection

A total of 604 records were identified through electronic database searches. After removal of duplicates (*n* = 138) using the Rayyan^®^ platform, 466 titles and abstracts were screened. Of these, 396 records were excluded at the title and abstract stage. A total of 70 articles were assessed for eligibility, with one additional article sought for retrieval. Of the articles reviewed, 47 were excluded for the following reasons: study type (*n* = 11), population (*n* = 13), concept (*n* = 21), or other reasons (*n* = 2), including a study [16] for which the full text could not be retrieved despite attempts to contact the corresponding author. This resulted in 23 studies being included in the final review. The selection process is illustrated in Figure 2, presented as a PRISMA 2020 flow diagram adapted for rapid scoping reviews.

### 3.2. Study Characteristics

The 23 included studies were published between 2014 and 2025. In total, 19 of these studies were conducted in sub-Saharan Africa (82.6%), with South Africa accounting for the largest share (*n* = 7), followed by Tanzania [17,18], Uganda [19,20], Zimbabwe [21,22], and one study each from Ghana [23], Malawi [24], Botswana [25], and Ethiopia [26]. One study was conducted in Tanzania and Uganda simultaneously [27]. Five studies were conducted in the United States (21.7%) [28,29,30,31,32].

Regarding the population, all studies included adults living with HIV (≥18 years) with at least one NCD. Sample sizes varied considerably, ranging from 8 to 1283 participants. Among studies that reported sex distribution (*n* = 20, 87%), female participants predominated, representing 73.2% of the total sample. Three studies enrolled exclusively female samples [25,30,33], reflecting their focus on specific conditions such as breast cancer and HIV care during pregnancy. Sex distribution was not reported in three studies.

Regarding the level of care, most studies were conducted in primary healthcare settings (*n* = 13, 56.5%), where HIV and NCD services are more frequently co-located. Hospital-based studies accounted for six studies, community-based settings for three, and one study was conducted in a home care context. One study [18] spanned secondary and tertiary care levels.

In terms of methodological design, qualitative approaches predominated (*n* = 11, 47.8%), including phenomenological, pragmatic, and focus group methodologies, reflecting the emphasis on understanding experiences, barriers, and facilitators to HIV-NCD integrated care. Cross-sectional studies accounted for six studies, observational cohort designs for three [19,32,34], and one prospective cohort [27], one longitudinal qualitative [20], and one quasi-experimental [32] implementation study were also included. The characteristics of all included studies are presented in Table 1.

**Table 1:** Summary of the main characteristics of the included studies.

Author, Year	Country	Objective	Study Design	Sample (*n*)	Sex (M/F)	Age Range	Care Level
Gooden et al., 2023 [17]	Tanzania	To understand the barriers and facilitators for prevention, early diagnosis and safe effective care for diabetes and hypertension within the current model of healthcare delivery among PLWH in Central Tanzania.	Pragmatic qualitative	36	14/22	20 to ≥61 years	Primary and secondary care
Ottaru et al., 2024 [18]	Tanzania	To describe the lived experiences, challenges, and coping strategies of adults living with HIV (ALHIV) for accessing care for hypertension and/or diabetes in HIV care and treatment clinics (CTCs) and other healthcare settings in Dar es Salaam, Tanzania.	Cross- sectional	33	15/18	34 to >73 years	Secondary and tertiary care
Low et al., 2019 [19]	Uganda	To assess the barriers in the care cascade for patients with HIV and cancer comorbidity.	Observational cohort	100	48/52	Median = 41 years	Hospital care
Bukenya et al., 2022 [20]	Uganda	To evaluate the integration of vertical health services for HIV, diabetes, and hypertension, offered in a feasibility study across five health units in Uganda.	Longitudinal qualitative	31	9/22	Mean ± SD: 45.1 ± 13.81	Primary health care
Chireshe et al., 2024 [21]	Zimbabwe	To explore barriers and facilitators to the provision of care to the patients with HIV and T2DM comorbidity.	Cross- sectional	8	NR	38 to 57 years	Primary health care
Chireshe et al., 2025 [22]	Zimbabwe	To identify areas for improvement in service delivery, ultimately fostering a more patient-centered approach to care that can enhance health outcomes for this vulnerable population.	Cross-sectional, descriptive, qualitative	20	5/15	18 to 75 years	Primary health care
Owusu et al., 2024 [23]	Ghana	To explore policy interventions aimed at improving the quality of life of HIV patients with hypertension or diabetes.	Qualitative descriptive design using a phenomenology approach	11	5/6	NR	Hospital care
Pfaff et al., 2017 [24]	Malawi	To assess the capacity of ART sites to provide care for hypertension and diabetes in rural Malawi.	Cross- sectional	25	NR	NR	Primary health care
Martei et al., 2023 [25]	Botswana	To evaluate patient-reported socioeconomic and cultural factors associated with adherence to guideline-concordant breast cancer therapy as planned, and how this may differ for PWH.	Qualitative using the Theory of Planned Behavior	10	All women	NR	Hospital care
van Koeveringe et al., 2023 [26]	Ethiopia	To create an understanding of the fundamental issues underlying comorbid care for ageing PLHIV from the perspective of people dealing and living with HIV, to inform health interventionists and public policy makers on optimising health care delivery.	Qualitative phenomenological	15	6/9	50 to 73 years	Hospital care
Namakoola et al., 2024 [27]	Tanzania and Uganda	To evaluate rates of retention in care and clinical control of hypertension, diabetes and HIV among participants receiving care from integrated care clinics for a period of up to 24 months in primary healthcare services in East Africa.	Prospective cohort	1283	353/930	Mean ± SD: 51.4 ± 11.7	Primary health care
Burkhalter et al., 2014 [28]	USA	To develop educational and cancer prevention and control interventions that build the capacities of AIDS service organizations to deliver evidence-based cancer programming to their PLWH clients or those at risk for HIV infection.	Cross-sectional, descriptive, qualitative	13	8/5	Mean ± SD: 42.8 ± 11.1	Community-based
Henry et al., 2023 [29]	USA	To examine oncologists’ knowledge, attitudes, and practices that influence cancer treatment decision-making.	Qualitative semistructured interviews	25	10/14 NR = 1	30 to 69 years	Hospital care
Warren-Jeanpiere et al., 2014 [30]	USA	To add to the literature by describing how age identity, co-morbidities, social responsibilities, and relationship status of older HIV-positive African American women influence their HIV self-management.	Qualitative using the focus group methodology	23	All women	52 to 65 years	Community-based
Webel et al., 2020 [31]	USA	To examine the perspectives of PLWH and their healthcare providers on how healthcare financing influences cardiovascular disease prevention provided in HIV and primary care clinics.	Qualitative	51	34/17	NR	Primary care clinics
Cutshaw et al., 2024 [32]	USA	To report details of the AAIM-High 12-month quasi-experimental implementation study, with specific focus on the co-primary effectiveness and implementation outcomes.	12-month single-arm hybrid type 2 effectiveness implementation	74	48/25 Trans woman = 1	Mean ± SD: 56.3 ± 10.8	Home care
Clouse et al., 2019 [33]	South Africa	To identify facilitators and barriers to follow-up engagement and treatment adherence.	Qualitative	25	All women	NR	Primary health care
Gausi et al., 2021 [34]	South Africa	To investigate the long-term patient outcomes among PLHIV with MM attending an IC model of care since implementation in Cape Town.	Observational retrospective cohort	247	59/188	Mean ± SD: 46.7 ± 8.6	Primary care clinics
Godongwana et al., 2021 [35]	South Africa	To investigate the challenges faced by health care providers (HCPs) in delivering the outcomes of the ICDM model, particularly, to patients living with the comorbidity of HIV and hypertension or diabetes, and to provide the perspectives of persons living with these conditions to understand their challenges.	Qualitative phenomenological	12	1/11	30 to 60 years	Primary health care
Ameh, 2020 [36]	South Africa	To determine the quality of care provided in the integrated model in 2013, describe patients’ and operational managers’ perceptions of quality of care in the integrated model in 2013, and assess effectiveness of the integrated model in controlling CD4 counts (>350 cells/mm^3^) and blood pressure (<140/90 mmHg) of patients from 2011 to 2013.	Cross- sectional	878	147/731	18 to ≥60 years	Primary health care
Johnson et al., 2024 [37]	South Africa	To identify context-specific facilitators of and barriers to hypertension care from the perspective of clinic managers, staff, and patients with the goal of informing the design of implementation strategies to address these.	Cross- sectional formative	46	17/29	Mean ± SD: 50 ± 8.5	Primary care clinics
Knight et al., 2018 [38]	South Africa	To explore the challenges of navigating healthcare for older persons living with HIV and NCD co-morbidity in two urban communities on the outskirts of Cape Town, South Africa, examining how healthcare-seeking experiences of older persons living with HIV may contribute to exacerbating the HIV-NCD syndemic.	Qualitative	23	13/10	50 to ≥65 years	Primary health care
Rajagopaul et al., 2025 [39]	South Africa	To explore the perceptions of healthcare workers regarding the quality of care provided to patients living with HIV and NCDs (diabetes mellitus and hypertension) in an urban district hospital in KwaZulu-Natal, South Africa, identifying the care model implemented and the facilitators and barriers to integrated care.	Cross- sectional	15	NR	NR	Hospital care

*n* = sample size; M: male; F: female; NR: not reported; SD: standard deviation.

### 3.3. Care Models, Integration Strategies, Challenges, and Outcomes

Table 2 summarizes the care models, integration strategies, and key challenges and outcomes identified across the 23 included studies.

Regarding the NCDs investigated, hypertension was the most frequently addressed condition, reported in 17 studies (73.9%), either in isolation or in combination with other conditions. Diabetes mellitus was examined in 14 studies (60.9%), often co-occurring with hypertension. Cancer was the focus of five studies, while cardiovascular disease was addressed in one study in the context of multiple comorbidities.

In terms of care models, integrated care was the most commonly described approach, identified in 10 studies (43.5%), whereas fragmented or siloed care was reported in eight studies. Two studies directly compared integrated and fragmented models, one described a bifurcated care model, and two did not specify the care model adopted. With respect to integration strategies, a range of approaches was reported, including support groups and home visits [23], health education sessions [18], community support groups [22], the Integrated Chronic Disease Management (ICDM) model [35], adherence clubs [34] and a virtual nurse-led follow-up intervention [32].

**Table 2:** Summary of the main characteristics of the care models, integration strategies, and key challenges and outcomes in the HIV-NCD syndemic.

Author, Year	NCDs Investigated	Care Model/Integration Strategies	Main Challenges of the HIV-NCD Syndemic	Main Results
Gooden et al., 2023 [17]	HTN, DM	Fragmented care/ NR	Fragmented services; lack of protocol for NCD screening; lack of access to diagnostic equipment; lack of continuity of NCD care; poverty; mental health problems among PLHIV; HIV stigma; lack of knowledge about NCDs among PLHIV and healthcare professionals.	Organisational/healthcare system factors: fragmented HIV and NCD services, no protocols on NCD screening; individual factors: HCPs’ knowledge of NCDs (for early diagnosis), HCPs’ personal practice (for early diagnosis and safe effective care); syndemic factors: poverty of PLHIV (barrier for prevention, early diagnosis and safe effective care), HIV stigma (barrier for early diagnosis and safe effective care), and mental health of PLHIV (barrier for prevention).
Ottaru et al., 2024 [18]	HTN, DM	Integrated care/ health education sessions	Drug and diagnostic material shortages for DM and HTN prior to integration; faulty or missing diagnostic equipment; financial barriers to transportation to health facilities; poverty and food insecurity affecting treatment adherence; inability to afford private clinical investigations or purchase drugs from private pharmacies prior to integration.	All participants reported shortages of diabetes and hypertension drugs and diagnostic equipment prior to the establishment of the integrated clinics; these were mostly addressed through the buffer stock; integration did not affect the already good provision of antiretroviral therapy; the cost of transport was reduced because of fewer clinic visits after integration; almost all DM and HTN users reported that drug shortages had become rare since the establishment of the integrated clinic; most participants observed that the integrated clinic reduced feelings of stigma for those living with HIV, as it was hard to tell what condition a person was being treated for.
Low et al., 2019 [19]	Cancer	Fragmented care/ NR	Difficulty traveling to multiple clinics/hospitals; conflicts between HIV and cancer appointments; prohibitive treatment costs; difficulty adhering to the quantity of medications/high pill burden; HIV stigma; cancer symptoms/illness limiting travel to HIV clinic.	Median time from first cancer symptoms to initiation of cancer care: 209 days (IQR 113–365); appraisal/behavioral delay (symptoms to first seeking care): median 31 day; diagnostic delay (first seeking care to cancer diagnosis): median 48.5 days; scheduling/referral delay: median 0.5 days; treatment delay (referral to Uganda Cancer Institute to initiating care): median 15 days; persons previously established in HIV care had shorter total cascade time (*p* = 0.04), shorter appraisal/behavioral delay (30 vs. 75 days; *p* = 0.02), and shorter diagnostic delay (44 vs. 117 days; *p* = 0.048).
Bukenya et al., 2022 [20]	HTN, DM	Fragmented care/ NR	Limited availability of DM screening at HIV CTCs; inconsistency in blood pressure measurement at HIV CTCs (malfunctioning machines); lack of anti-hypertensives and diabetes medication at HIV CTCs; lack of formal referral to NCD clinics; uncoordinated and fragmented healthcare delivery system.	The majority of participants (*n* = 23) were not currently attending any clinics for HTN/DM management at the time of the study; HIV CTCs at regional referral hospitals provided HTN screening more consistently than district hospitals and health centers; none of the participants reported having their blood sugar measured at the HIV CTC; none of the participants reported undergoing screening for DM symptoms at the HIV CTCs.
Chireshe et al., 2024 [21]	DM	Fragmented care/ NR	Chronic shortage of healthcare providers; lack of training and absence of updated guidelines; unavailability of essential medicines and supplies; inadequate laboratory infrastructure.	Patients with comorbidities frequently miss DM appointments due to separate appointment schedules and costs; counselling for diabetes patients only occasionally provided due to staff shortages; absence of guidelines for diabetes management in several facilities; primary care facilities in Harare scored below the 90% readiness target, indicating inadequate preparedness to care for patients with HIV and T2DM comorbidity.
Chireshe et al., 2025 [22]	DM	Integrated vs. fragmented care/ community support groups	Polypharmacy and a high number of pills in continuous use; negative effects on mental health.	The fragmented care model resulted in multiple clinic visits and consultations with different professionals, as well as a lack of confidentiality due to the segregation of HIV-positive patients in separate environments. This model also increased financial costs related to transportation and absenteeism from school and work.
Owusu et al., 2024 [23]	HTN, DM	Integrated care/support group system; home visits	Non-adherence to medication, stigma, cost of NCDs medications, accessibility issues to NCDs services.	Support groups improved psychological well-being and treatment adherence among PLHIV and comorbidities; home visits helped monitor patients’ living conditions and adherence to treatment.
Pfaff et al., 2017 [24]	HTN, DM	Integrated vs. fragmented care/ NR	Ongoing drug and equipment shortages for NCD management at all facility levels.	NCD care was predominantly delivered at the central referral hospital; health centres provided almost no NCD care; in February 2014, only 943 people received treatment for hypertension and 310 for diabetes at the study sites, representing an estimated 1.5% and 2.7% of the estimated disease burden respectively; 60% of hospitals had at least one clinician and one nurse trained in NCD care; only 5% of health centres had a trained clinician and 8% had a trained nurse; 100% of hospitals and 80% of health centres had at least one blood pressure machine; 80% of hospitals and 32% of health centres had a glucometer.
Martei et al., 2023 [25]	Cancer	Fragmented care/ NR	Intersectional stigma of both HIV and breast cancer; therapy-related toxicity because of administration of both cancer-directed therapy and HIV treatment; parallel care systems; challenges coordinating appointments for HIV–Cancer.	Integrated or a simplification of their HIV and cancer regimens as facilitators associated with treatment fidelity; PLHIV felt empowered about management of their cancer because of prior success in managing their HIV.
van Koeveringe et al., 2023 [26]	HTN, DM	Fragmented care/ NR	Continuous polypharmacy causing fatigue in people living with HIV; difficulties in prescribing ART and concomitant medications (risk of side effects and drug interactions); difficulties in traveling between different appointments in different locations.	Providers did not provide with enough information on how to manage multiple conditions alongside their HIV; deficiency of the providers due to lack of training in geriatrics and non-AIDS-related conditions; providers as key enablers of good management of their illnesses; many patients experienced difficulties in establishing trusting relationships with their physicians; the distant relationship was mainly attributed to the fragmentation in care; frustration with the referral system: long waiting times, under-resourced teams, or unavailability of specialists; due to the lack of standardisation of geriatric assessments, patients are often diagnosed when the comorbidity is already in a progressed stage; the main barrier to accessing healthcare is the cost of treatment, especially medications for NCDs.
Namakoola et al., 2024 [27]	HTN, DM	Integrated care/ integrated care clinics	Lack of funding for HTN and DM medications; inability to perform regular monitoring; low adherence and counseling regarding lifestyle changes; lack of trained healthcare professionals.	The integrated care model can achieve high rates of retention in care long-term in primary healthcare settings. Furthermore, the model does not adversely impact HIV care considering that more than 90% of people with HIV had viral suppression. However, the control of glycaemia and blood pressure among participants living with diabetes and hypertension remained low.
Burkhalter et al., 2014 [28]	Cancer	Integrated care/ NR	Limited experience with cancer-focused programs among AIDS service organizations; lack of funding and resources to implement cancer services; nonengagement in HIV medical care leading to later-stage cancer diagnoses.	Most agencies had limited experience with cancer-focused programs, and when they had, programs were not framed as cancer-specific; agencies need resources and collaborative partnerships to effectively incorporate cancer services; staff and clients must be educated about the relevance of cancer to HIV/AIDS.
Henry et al., 2023 [29]	Cancer	Fragmented care/ NR	Lack of formal training among oncologists in treating PLHIV with comorbid malignancies; fear of inadvertent disclosure of HIV status during clinical encounters, particularly in the presence of family members or caregivers; hesitancy to discuss HIV status directly with patients due to confidentiality concerns, restricting important clinical discussions.	All 25 (100%) oncologists reported having no formal training or coursework on HIV malignancies; 21 (84%) raised concerns about patient confidentiality and fear of inadvertent HIV disclosure; 17 (68%) discussed cancer treatment preferences and attitudes toward PLHIV; 23 (92%) reported communicating with ID providers to facilitate cancer care; 22 (88%) noted collaboration with other specialists (social workers, pharmacists, etc.).
Warren-Jeanpiere et al., 2014 [30]	HTN, DM, cancer, heart disease	NR	Being single and lonely contributes to the difficulty of managing their HIV; lack of income and health insurance; the effects of medication often conflict with the very structure of paid work.	The ability and desire to self-manage health increased with age; managing HIV and comorbid conditions while aging requires considerable negotiation on a daily basis; companionship received from male partners serves to inspire them to self-manage their HIV and other co-morbid conditions; self-management of HIV and co-morbid conditions is facilitated by support from intimate others; comorbidities require more effort to control than HIV.
Webel et al., 2020 [31]	HTN	Integrated care/ NR	Limited time with the healthcare professional during appointments; high treatment costs and difficulty in obtaining coverage from health insurance plans; lack of funding.	Health insurance payers have substantial control over decisions affecting the cardiovascular care and treatment of PLHIV; insurance regulations are not tailored for PLWH who are at increased risk for cardiovascular diseases; the grant-funded programs were seen as mostly beneficial to participant’s cardiovascular health because they opened up new opportunities for patients but had several negative consequences (e.g., shaping the program to the funder’s priorities, increased workload) that may limit their impact; limited time with the healthcare professional during appointments makes it difficult to address multiple chronic diseases, build rapport, and provide counseling.
Cutshaw et al., 2024 [32]	HTN	Integrated care/ virtual follow-up	Deficiencies in atherosclerotic cardiovascular disease risk factor management, including perceived lack of expertise and competing time demands during HIV clinical encounters.	The study effectively improved hypertension control in PLHIV through a virtual, nurse-led intervention; over 12 months, the average patient-performed home systolic blood pressure decreased by 7.7 mmHg; the percentage of patients at treatment goal increased from 46.0% to 72.5% at 12 months.
Clouse et al., 2019 [33]	HTN	Fragmented care/ NR	Lack of integration of HIV, NCD, and baby care services; visits often on different days, even when provided in the same clinic, requiring many trips; increased time commitment (median 3.25 h per NCD visit, 2.75 h for HIV, 1.5 h for baby’s visit); transportation and logistics barriers.	HIV and NCD visits usually occurred in the same clinic, but often on different days; baby care visits nearly always were on different days; women reported attending more visits for themselves during pregnancy than after; after delivery, focus shifted to the baby’s healthcare; disrespectful treatment by clinical staff was frequently noted, particularly related to HIV stigma.
Gausi et al., 2021 [34]	HTN, DM	Integrated care/ Adherence clubs	The absence of ongoing promotion of health related to NCDs in IC clinics to support the maintenance of positive behavioral changes.	Patients with a more recent diagnosis of NCDs showed better disease control compared to patients with an older diagnosis; women were more likely to control their NCDs compared to men; multimorbid PLHIV achieved high levels of HIV control; however, intensified interventions are needed to maintain NCD control in the long term.
Godongwana et al., 2021 [35]	HTN, DM	Integrated care/ Integrated Chronic Disease Management (ICDM) model	Segregation of PLHIV from other chronic patients; discrimination due to body changes; social non-acceptance of multiple illnesses; treatment fatigue, mainly induced by polypharmacy, medication side effects, and multiple appointments.	Lack of professionals in the units, which overburdens workers and slows down service delivery; unavailability of medications, especially for HTN and DM; lack of training for professionals to use the integrated model; patients who had not disclosed their serological status for HIV or other chronic diseases frequently abandon treatment; unemployment is one of the factors that hindered the self-management of chronic diseases, as it results in financial challenges that affected access to health services, food security, and the ability to follow treatment properly.
Ameh, 2020 [36]	HTN	Integrated care/ NR	HIV stigma in communities; staff shortage; anti-hypertension drug stock-outs; malfunctioning or unavailable blood pressure machines; dysfunctional pre-packing of drugs.	Operational managers were satisfied with 16 of the 17 dimensions of care; patients reported satisfaction with 14 dimensions; both patients and operational managers reported low satisfaction with patient waiting time; patients expressed dissatisfaction with defaulter tracing activities (29%) and clinic appointments (20%); integration of HIV and NCD services was associated with HIV stigma reduction due to non-segregation of patients.
Johnson et al., 2024 [37]	HTN	Integrated care/ integrated care clubs	Clinics with limited structural and operational capacity to support the implementation of integrated care models; education and training on chronic care guidelines are inconsistent and often insufficient across clinics; lack of resources and fragmented clinical workflow; lack of awareness about the risk of hypertension, fear, and frustration.	High adherence to treatment with the integrated model (>94%); high viral suppression (>99%); high retention without care, with 93.1% retained in care and 6.9% lost to follow-up; limited/inconsistent control of NCDs, with controlled blood pressure in less than 50% of cases and glycemic control with initial improvement and decline after 12 months; high levels of HIV control; however, control of NCDs was not ideal.
Knight et al., 2018 [38]	HTN, DM	Bifurcated and siloed care/Chronic Care Club	Bifurcated/siloed provision of HIV and NCD care; separate appointments for each condition, often on different days or resulting in scheduling conflicts; physical distance between facilities; financial barriers to transportation.	Respondents sought care and accessed treatment for both HIV and other chronic conditions, but these services were provided at different health facilities or by different health providers; the siloed provision of HIV and NCD care meant that services were not integrated and providers were often unaware of patients’ other conditions; tuberculosis and HIV treatment were integrated, but other NCDs were not; older persons experienced significant physical, financial, and logistical barriers to accessing bifurcated care.
Rajagopaul et al., 2025 [39]	HTN, DM	Integrated care/ NR	Fragmented care; staff shortages; high patient loads; long waiting times; inadequate and inconsistent staff training for NCD management; equipment shortages.	Most participants reported that HIV and NCDs were diagnosed, investigated and managed at the hospital; 10 (67%) participants reported that patients were referred to Outpatient Department for NCDs, highlighting fragmented care; healthcare professionals welcomed integrated HIV–NCD care, recognising its potential to reduce stigma, improve continuity, and enhance patient outcomes; integrated care was perceived as beneficial but constrained by shortages of medicines, staff, and training opportunities.

NR: not reported.

The main challenges identified were consistent across settings and included staff shortages, gaps in drug and equipment supply, high patient loads, limited training for NCD management within HIV services, HIV-related stigma, and financial barriers to accessing care. Fragmented care was frequently associated with multiple appointments on different days, long travel distances, and increased costs for patients. In contrast, studies conducted in integrated care settings reported improvements in treatment adherence, reduction in stigma, and better retention in care. However, sustained control of NCDs, particularly hypertension and diabetes, remained limited across most contexts.

### 3.4. Nursing Practice

Eight of the 23 included studies reported data on nursing practice (34.8%) [17,21,22,23,27,32,36,37]. Clinical service delivery was the predominant nursing role across all eight studies. Additional roles included counselling, home visits, and support group facilitation [23], facility management [21], participation in integrated care implementation processes [37], and care coordination, medication protocol management, and blood pressure monitoring within a virtual nurse-led intervention [32].

### 3.5. Patients’ Perceptions

Patients’ perceptions of nursing care were reported in four studies (Table 3). Positive perceptions included satisfaction with nurses’ clinical competence, friendliness, and communication skills [36], the value placed on continuity of care with familiar providers [17], and recognition of compassion and cultural sensitivity as essential qualities [22]. Negative perceptions included work-related exhaustion leading to unprofessional conduct [36], patients feeling disempowered from decisions about their own care [22], inadequate education on NCDs prior to diagnosis [17], and a desire for improved communication from healthcare professionals [37].

**Table 3:** Summary of patients’ perceptions of the included studies.

Author, Year	Patients’ Perceptions of Nursing Care
Gooden et al., 2023 [17]	Provided encouragement and education that gave patients hope; Educated on diet and exercise, but not adequately on NCDs prior to diagnosis; Valued continuity of care with familiar providers.
Chireshe et al., 2025 [22]	The compassion and cultural sensitivity among nurses; patients feel disempowered for not participating in discussions about their care; patients seek compassionate caregivers who speak kindly, listen, and answer questions honestly.
Ameh, 2020 [36]	Patients reported that work-related exhaustion of nurses led to ‘complicated’ behaviour negatively impacting quality of care; Felt unsafe due to unprofessional conduct: a nurse was observed sending patients or cleaners to fetch medicines, raising fears of medication swapping; Satisfaction with nurses’ competencies (87.6%) and confidence in nurses (85.5%) in the quantitative study; friendliness (92.4%) and professional conduct (86.2%) of nurses; coherence of care (97.4%) and communication (98.9%).
Johnson et al., 2024 [37,38]	Patients reported that getting to know the professionals is positive, but that the professionals should improve their communication skills.

### 3.6. Implications for Care

The main implications for care converged around the need to strengthen integrated HIV-NCD service delivery, expand counselling [23] and health education [17,22], ensure consistent supply of medications [21,36] and functional equipment [36], address staff shortages [23], reduce HIV-related stigma [17,22,36], and invest in innovative models such as telehealth [22] and virtual nurse-led follow-up [32] (Table 4).

## 4. Discussion

The interpretation of the findings was guided by an analytical framework that articulates biological, structural, and organizational dimensions, enabling the HIV-NCD syndemic to be understood beyond the mere coexistence of diseases, as a complex phenomenon produced by the interaction between multimorbidity, social vulnerabilities, and the ways in which health systems organize care.

### 4.1. Fragmentation of Care in the Context of the Complexity of the HIV-NCD Syndemic

The coexistence of HIV and NCDs has generated an increasingly prevalent scenario of multimorbidity, particularly as advances in antiretroviral therapy prolong the survival of PLHIV, resulting in premature aging and an increased burden of associated chronic conditions. In this context, the findings of this review indicate that health systems continue to operate predominantly under a biomedical logic centered on isolated diseases, which translates into siloed care organization, with distinct care pathways for HIV and NCDs [17,22,38]. This fragmentation undermines the comprehensiveness of care and reveals a disconnect between the complexity of users’ needs and the way services are structured.

From an organizational perspective, fragmentation manifests in the requirement for multiple consultations, often scheduled on different days and at different locations, with limited coordination among professionals and services [19,33]. Studies included in this review demonstrate that this logic results in delays in diagnosis and initiation of NCD treatment, as well as increased burden on users, who face logistical, financial, and emotional challenges in maintaining continuity of care [18,19]. Furthermore, the lack of integration between services contributes to gaps in clinical communication and fragmentation of therapeutic decision-making, potentially compromising the safety and effectiveness of care [37,39].

Frequent travel requirements, transportation costs, and incompatibility between care schedules emerge as concrete barriers to access, particularly in contexts of greater social vulnerability [20,22]. Difficulties related to health insurance coverage have also been reported by PLHIV, especially in countries with private insurance-based systems, such as the United States [30,31].

Although fragmentation of care appears as a cross-cutting finding, its manifestations vary according to service organization and local contexts. In urban settings in South Africa, for example, fragmentation occurs across geographically distinct units, requiring movement between specialized services [38], whereas in Tanzania it is expressed through administrative separation within the same care complex, such as between HIV clinics and NCD outpatient services [17]. In addition, evidence of hybrid models, in which integrated and fragmented practices coexist, suggests that fragmentation is not a fixed state but rather an organizational continuum influenced by structural and institutional conditions [22].

Beyond organizational dimensions, the findings reinforce that fragmentation cannot be understood in isolation from the syndemic determinants that shape the experience of living with HIV and NCDs. Factors such as poverty, stigma, and psychological distress not only hinder access to and adherence to services but also interact synergistically, increasing individual vulnerability and worsening health outcomes [17,35].

HIV-related stigma, for example, may lead to avoidance of services or non-adherence to treatment, while economic insecurity directly affects the ability to follow complex therapeutic regimens [20,23]. Moreover, the overlap of chronic conditions and social demands intensifies mental distress and treatment burden, creating a context in which users’ needs exceed the scope of fragmented care models [26].

From a syndemic perspective, fragmentation of care functions not only as an organizational barrier but also as an element that interacts synergistically with structural vulnerabilities, intensifying the effects of multimorbidity. The segregation of services may reinforce HIV-related stigma, leading to discontinuity of care, diagnostic delays, and worsening of chronic conditions, thereby creating a feedback loop between service organization and social determinants. In this sense, care integration should be understood not merely as a service strategy but as a structural intervention capable of challenging this dynamic.

### 4.2. Care Integration as a Strategy: Advances, Limits, and Structural Determinants

In light of the limitations of fragmented models, care integration emerges as a central strategy to address the complexity of the HIV-NCD syndemic, proposing the reorganization of services through more coordinated, continuous, and person-centered approaches. The studies analyzed highlight the implementation of different organizational arrangements, including integrated HIV-NCD clinics, support groups, adherence clubs, health education-based interventions, and home visits [20,23,34], aiming to overcome siloed care and strengthen coordination between services, particularly within primary health care.

The findings indicate important benefits, such as improved treatment adherence, increased retention in care, and greater continuity of services [27,34], as well as reduced HIV-related stigma, facilitated by the non-segregation of users into disease-specific services [20,36]. Community-based strategies also demonstrate potential to strengthen self-care and social support [22,28,30].

However, despite these advances, integration presents important limitations. Clinical control of NCDs remains suboptimal even within integrated models [27,34], indicating that service reorganization alone does not guarantee improved health outcomes. The persistence of these findings suggests that integration, when implemented without strengthening social support networks, food security, and continuity in the provision of supplies, may result in a reorganization that shifts, but does not eliminate structural barriers. In contexts of chronic underfunding, integration tends to redistribute the burden of care without addressing the determinants underlying the complexity of the syndemic.

Moreover, the implementation of integrated models often occurs in contexts of limited human resources, insufficient medication supply, and inadequate professional training [35,37], compromising the quality of care. Therefore, integration should be understood as a process conditioned by structural and social determinants, implying that integration policies must be accompanied by sustained investments in infrastructure, supply provision, and workforce development; otherwise, they risk reproducing, in a new format, the same barriers they seek to overcome.

### 4.3. Nursing as a Structural Axis of Care Integration

From a syndemic perspective, nursing occupies a strategic position by operating at the interface between the biological, structural, and organizational dimensions of care. At the biological level, nurses are involved in the management of multimorbidity, polypharmacy, and the promotion of treatment adherence; at the structural level, they mediate the relationship between social vulnerabilities and access to services; and at the organizational level, they play a central role in care coordination and in linking different levels of care. This boundary position makes nursing a key element for integration, but also a point of tension when health systems fail to provide adequate support. In this context, the studies analyzed show that nurses act strategically in coordinating across levels of care, managing cases, and providing longitudinal follow-up, thereby contributing to continuity and comprehensiveness of care [23,37].

However, this role does not manifest uniformly. In primary health care, nursing practice is primarily focused on care coordination and longitudinal follow-up, whereas in hospital or specialized settings it involves the management of polypharmacy and coordination across levels of care, requiring different competencies and carrying implications for professional training.

In addition, nursing practice encompasses a broad range of activities, including health education, support for self-care, home-based follow-up, and psychosocial support, elements that are fundamental for addressing barriers associated with the syndemic [17,22]. The establishment of therapeutic relationships emerges as a key component of care quality [22].

In this sense, the analysis of the included studies allows us to identify that nursing practice in integrated care models is operationalized through a set of interventions that articulate clinical, educational, and psychosocial dimensions of care. At the clinical level, key actions include care coordination and case management, with longitudinal follow-up of users and articulation across different levels of care, thereby promoting continuity of care and service integration [23,37].

In the educational domain, nurses provide guidance on lifestyle modifications, polypharmacy management, and differentiation between medications for HIV and NCDs, using strategies such as individual and group counseling during consultations [17,35]. In addition, they play a role in coordinating care by aligning appointments and facilitating continuity of care, particularly within primary health care settings [37,39].

Psychosocial and community-based interventions are also frequently led by nurses, including support groups, home visits, and longitudinal follow-up of individuals with multimorbidity [20,23]. These actions strengthen therapeutic relationships, reduce stigma, and extend care beyond the clinical setting by incorporating social and territorial dimensions that are essential for addressing the HIV–NCD syndemic. In resource-limited settings, nurse-led virtual interventions, such as remote monitoring and teleconsultations, have shown to be feasible strategies to expand access and improve clinical outcomes [32].

Users reported mixed perceptions of HIV and NCD services. While they recognized nurses’ competence, interpersonal skills, communication, and sensitivity, as well as the value of encouragement, education, and continuity of care, they also identified significant shortcomings, including insufficient guidance on NCDs, fragmented care, barriers to access, and insecurity related to unprofessional conduct associated with staff burnout [17,36,37]. In addition, users reported feelings of disempowerment due to limited participation in decision-making and challenges in self-management, highlighting the need to strengthen more integrated, participatory, and patient-centered care approaches [22].

Nevertheless, nursing practice is constrained by structural limitations such as workload burden, workforce shortages, and lack of specific training [21,35]. These conditions reveal a central contradiction: nurses are simultaneously the primary agents of integration and the group most exposed to the structural weaknesses of health systems.

### 4.4. Limitations

From a rapid scoping review perspective, this study presents limitations inherent to its methodology, particularly due to the adoption of strategies prioritizing feasibility and timeliness. These include restrictions regarding the publication period (2010–2026), language (English, Spanish, and Portuguese), inclusion of peer-reviewed publications only, and the exclusion of grey literature. Such decisions may have limited the identification of materials addressing the implementation of integrated care models in practice, such as manuals, guidelines, and related documents.

In addition, no formal methodological quality appraisal or risk of bias assessment was conducted, in line with scoping review methodology, which limits the ability to evaluate the robustness of the included evidence. The heterogeneity of study designs, populations, and healthcare contexts also restricts the comparability of findings and the generalizability of results. Furthermore, the predominance of studies conducted in sub-Saharan Africa may limit the applicability of findings to other settings. Potential publication bias and selection bias during the screening process cannot be ruled out. Finally, although a syndemic perspective informed the analytical interpretation, the inconsistent use of this concept in primary studies may have influenced the synthesis of findings.

### 4.5. Strengths

This study has several notable strengths that enhance the robustness and relevance of its findings. First, it follows a rigorous methodological framework aligned with established scoping review guidelines, ensuring transparency, reproducibility, and methodological consistency throughout all stages of the review process. The comprehensive search strategy, conducted across multiple databases, increases the likelihood of capturing a wide spectrum of relevant evidence and reduces the risk of selection bias.

In addition, the inclusion of heterogeneous study designs represents a key strength, as it enables a more nuanced understanding of the multifaceted relationship between HIV and NCDs, encompassing epidemiological, clinical, and contextual dimensions. The use of a structured and systematic data extraction and synthesis approach further strengthens the reliability of the findings by allowing the identification of consistent patterns, research gaps, and emerging trends.

Importantly, this review advances the current body of knowledge by moving beyond isolated analyses of HIV or NCDs and instead providing an integrated perspective on multimorbidity, an area that remains underexplored in the literature. By doing so, it contributes to a more comprehensive understanding of the epidemiological burden and complexity of HIV-NCD interactions. Finally, the identification of critical knowledge gaps offers valuable directions for future research and supports the development of more targeted and evidence-informed public health and clinical strategies.

## 5. Conclusions

The present scoping review demonstrated that the organization of care in the HIV-NCD syndemic remains predominantly characterized by fragmented models, which are insufficient to address the complexity of multimorbidity. In this context, integrated care models emerge as a promising strategy; however, their effects remain limited in settings marked by health inequalities, such as lack of access to services and essential inputs, poverty, and food insecurity. The findings also highlight the central role of nursing as a structural axis of integrated models, particularly given its coordinating potential and its proximity to communities and to the living conditions of users within primary health care. Nevertheless, this centrality contrasts with often precarious working conditions, revealing a tension between the responsibilities assigned to these professionals and the support effectively provided by health systems.

In this context, the development of effective responses to the syndemic requires more than organizational innovation. It demands sustained political commitment and structural investment, including expansion of the health workforce, assurance of supplies for NCD care, improvement of service infrastructure, and policies aimed at professional recognition and support. Finally, this review underscores the importance of analytical and intervention approaches that consider the HIV-NCD syndemic in its full complexity, articulating biological, structural, and organizational dimensions. Future studies should advance the production of more robust evidence, including longitudinal and cost-effectiveness analyses, and incorporate the perspectives of populations that are often marginalized within health systems.

## Figures and Tables

**Table 4:** Summary of implications for care of the included studies.

Author, Year	Main Implications for Care
Gooden et al., 2023 [17]	Targeted interventions within existing healthcare structures, improved health education on HIV-NCD comorbidity, and collaborative solutions for diagnostic and medication access are needed to enhance care quality; integration of HIV and NCD services, stigma reduction, and addressing syndemic factors such as poverty and mental health are essential to improve clinical outcomes and patient safety.
Chireshe et al., 2024 [21]	Improved resource allocation and multisectoral collaboration to improve the delivery of healthcare services; proactive recruitment and retention of staff, as well as active training of health providers to improve quality of care; enhanced distribution of resources to primary care facilities to close gaps in the supply of necessary medications and supplies; patient education to increase patients’ understanding of HIV and T2DM management and to encourage treatment adherence; training and support for healthcare professionals in managing HIV and T2DM.
Chireshe et al., 2025 [22]	All primary health care clinics should offer integrated services (HIV, NCD and mental health services); there is a need to upskill healthcare providers at clinics; use e-health or Telehealth Services to reduce waiting times and transport costs for patients; improve communication channels, strengthen health education workshops, and invest in research on the HIV-DM syndemic to improve public policies.
Owusu et al., 2024 [23]	Support groups should be strengthened to improve psychological well-being and treatment adherence among patients living with HIV and NCDs; home visits should be implemented to monitor patients’ living conditions and adherence to treatment; provision of free drugs is important to support continuity of treatment for people living with HIV and comorbidities; counselling services should be expanded to support coping, adherence, and self-management of chronic conditions; integrated care approaches should be strengthened to address HIV and NCD comorbidities simultaneously.
Namakoola et al., 2024 [27]	The need for integrated models that offer simultaneous care for HIV and NCDs; the need for intensified interventions to maintain control of NCDs; integration improves retention and adherence, but does not guarantee sustained control of NCDs, which requires awareness-raising for healthy habits.
Cutshaw et al., 2024 [32]	Care delivery strategies that increase access to comprehensive cardiovascular disease risk management are a priority; virtual, nurse-led intervention as a viable model to expand access to care; adaptation to a telemedicine-based strategy in the contemporary care context.
Ameh, 2020 [36]	The HIV programme needs to be more extensively leveraged for hypertension treatment; further strengthening of the broader health system in which the ICDM model is embedded is required to achieve optimal BP control; reduction in HIV stigma through integration of HIV and NCD services in the same consultation rooms may increase uptake of HIV services; addressing staff shortage is critical to reduce work overload and improve quality of nursing care in integrated settings; ensuring consistent supply of essential medicines (antihypertensives) and functional diagnostic equipment (BP machines) is necessary for effective care.
Johnson et al., 2024 [37]	The need for tailored implementation strategies for HIV-hypertension integration; strengthening structural and organizational resources; alignment between patient and professional perceptions to improve care.

## Data Availability

No new data were created.

## Supplementary Materials

The following supporting information can be downloaded at: https://www.mdpi.com/article/10.3390/ijerph23050642/s1, Table S1: Search strategies based on the database; Table S2: Additional information from the included studies; File S1: PRISMA checklist [40].

## Abbreviations

ALHIV	Adults living with HIV
ART	Antiretroviral Therapy
BP	Blood pressure
CD4	Cluster of Differentiation 4
CINAHL	Cumulative Index to Nursing and Allied Health Literature
CTCs	HIV care and treatment clinics
DeCS	Health Sciences Descriptors
DM	Diabetes mellitus
HCPs	Health care providers
HIV	Human Immunodeficiency Virus
HTN	Hypertension
IC	Integrated care
ICDM	Integrated Chronic Disease Management
IQR	Interquartile range
JBI	Joanna Briggs Institute
LILACS	Latin American and Caribbean Health Sciences Literature
MeSH	Medical Subject Headings
NCDs	Non-Communicable Diseases
PCC	Population, Concept, Context
PHC	Primary Health Care
PLHIV/PLWH/PWH	People Living with HIV
PRISMA	Preferred Reporting Items for Systematic Reviews and Meta-Analyses
PRISMA-ScR	Preferred Reporting Items for Systematic Reviews and Meta-Analyses Extension for Scoping Reviews
WHO	World Health Organization
