# Supplementary material for: Organization and Integration of Care in the HIV–Non-Communicable Disease Syndemic: A Rapid Scoping Review
Source: Int J Environ Res Public Health. 2026 May 12;23(5):642. doi: 10.3390/ijerph23050642 (PMC13207135; doi:10.3390/ijerph23050642)
Supplement: Supplementary file 1 [file ijerph-23-00642-s001.zip › ijerph-4278229-supplementary.pdf]

Table S1. Search strategies based on the database.

Database	Primary search strategy	Secondary search strategy
Pubmed	("HIV"[Mesh] OR "HIV Infections"[Mesh] OR "people living with HIV"[TIAB] OR PLHIV[TIAB] OR "HIV-positive"[TIAB]) AND ("Multimorbidity"[Mesh] OR "Comorbidity"[Mesh] OR "Chronic Disease"[Mesh] OR multimorbid*[TIAB] OR comorbid*[TIAB] OR "chronic disease*"[TIAB] OR "chronic condition*"[TIAB]) AND ("Delivery of Health Care, Integrated"[Mesh] OR "Continuity of Patient Care"[Mesh] OR integrated care[TIAB] OR care integration[TIAB] OR "models of care"[TIAB] OR "care coordination"[TIAB]) AND ("Primary Health Care"[Mesh] OR "Health Services Accessibility"[Mesh] OR health system*[TIAB] OR health service*[TIAB] OR "access to care"[TIAB])	("HIV"[Mesh] OR "HIV Infections"[Mesh] OR "people living with HIV"[TIAB] OR PLHIV[TIAB] OR "HIV-positive"[TIAB]) AND ("Multimorbidity"[Mesh] OR "Comorbidity"[Mesh] OR "Chronic Disease"[Mesh] OR multimorbid*[TIAB] OR comorbid*[TIAB] OR "chronic disease*"[TIAB] OR "chronic condition*"[TIAB]) AND ("Delivery of Health Care, Integrated"[Mesh] OR "Continuity of Patient Care"[Mesh] OR integrated care[TIAB] OR care integration[TIAB] OR "models of care"[TIAB] OR "care coordination"[TIAB]) AND ("Nursing"[Mesh] OR "Nursing Care"[Mesh] OR "Nurses"[Mesh] OR nurse* OR "nursing care" OR "nursing practice" OR "nursing role" OR "advanced practice nursing")
Scopus	TITLE-ABS-KEY (("people living with HIV" OR PLHIV OR "HIV-positive" OR HIV) AND (multimorbid* OR comorbid* OR "chronic disease*" OR "chronic condition*") AND ("integrated care" OR "care integration" OR "models of care" OR "care coordination") AND ("primary health care" OR "health system*" OR "health service*" OR "access to care"))	TITLE-ABS-KEY (("people living with HIV" OR PLHIV OR "HIV-positive" OR HIV) AND (multimorbid* OR comorbid* OR "chronic disease*" OR "chronic condition*") AND ("integrated care" OR "care integration" OR "models of care" OR "care coordination") AND (nurse* OR "nursing care" OR "nursing practice" OR "nursing role" OR "advanced practice nursing"))

CINAHL	((MH "HIV+") OR (MH "HIV Infections+") OR (MH "Acquired Immunodeficiency Syndrome+") OR TI "people living with HIV" OR AB "people living with HIV" OR TI PLHIV OR AB PLHIV OR TI "HIV-positive" OR AB "HIV-positive")<br>AND<br>((MH "Chronic Disease+") OR (MH "Comorbidity+") OR (MH "Multimorbidity+") OR TI "chronic disease*" OR AB "chronic disease*" OR TI multimorbid* OR AB multimorbid* OR TI comorbid* OR AB comorbid*)<br>AND<br>((MH "Delivery of Health Care+") OR (MH "Integrated Health Care Systems+") OR (MH "Continuity of Patient Care+") OR TI "integrated care" OR AB "integrated care" OR TI "care coordination" OR AB "care coordination" OR TI "care model*" OR AB "care model*")<br>AND<br>((MH "Health Services+") OR (MH "Primary Health Care+") OR (MH "Health Policy+") OR TI "health system*" OR AB "health system*" OR TI "service delivery" OR AB "service delivery")<br>AND<br>((TI qualitative OR AB qualitative OR TI interview* OR AB interview* OR TI "focus group*" OR AB "focus group*" OR TI "cohort stud*" OR AB "cohort stud*" OR TI "cross sectional stud*" OR AB "cross sectional stud*" OR TI "case control stud*" OR AB "case control stud*" OR TI "program evaluation" OR AB "program evaluation")<br>NOT PT Review<br>NOT PT "Clinical Trial"	((MH "HIV+") OR TI "people living with HIV" OR AB "people living with HIV")<br>AND<br>((MH "Chronic Disease+") OR TI multimorbid* OR AB multimorbid*)<br>AND<br>(TI "integrated care" OR AB "integrated care")<br>AND<br>((MH "Nursing+") OR (MH "Nurses+") OR TI nurse* OR AB nurse* OR TI "nursing practice" OR AB "nursing practice")

LILACS	(mh:(HIV OR "Infecções por HIV") OR tw:("people living with HIV" OR PLHIV OR "HIV-positive")) AND (mh:("Doença Crônica" OR "Multimorbidade" OR "Comorbidade") OR tw:(multimorbid* OR comorbid* OR "chronic disease*" OR "chronic condition*")) AND (mh:("Atenção Integral à Saúde" OR "Continuidade da Assistência ao Paciente") OR tw:("integrated care" OR "care integration" OR "models of care" OR "care coordination")) AND (mh:("Atenção Primária à Saúde" OR "Acesso aos Serviços de Saúde") OR tw:("health system*" OR "health service*" OR "access to care"))	(mh:(HIV OR "Infecções por HIV") OR tw:("people living with HIV" OR PLHIV OR "HIV-positive")) AND (mh:("Doença Crônica" OR "Multimorbidade" OR "Comorbidade") OR tw:(multimorbid* OR comorbid* OR "chronic disease*" OR "chronic condition*")) AND (mh:("Atenção Integral à Saúde" OR "Continuidade da Assistência ao Paciente") OR tw:("integrated care" OR "care integration" OR "models of care" OR "care coordination")) AND (mh:(Enfermagem OR Enfermeiros) OR tw:(enfermagem OR enfermeiro*))

Table S2. Additional information from the included studies.

ID	Eligibility criteria	Other results	Limitations
1	Inclusion criteria:<br>Healthcare professionals and support staff directly involved in the care and management of HIV patients;<br>Healthcare professionals with at least two years of experience in an ART unit.<br><br>Exclusion criteria:<br>Healthcare professionals with temporary contracts;<br>Individuals who declined to offer informed consent for participating in interviews.	Provision of free drugs supported continuity of treatment and improved patient support;<br>Counselling contributed to improved coping and adherence to treatment.	The study included a relatively small sample size of 11 participants from three regions in Ghana, therefore the findings may lack generalizability to a larger population.
2	Inclusion criteria:<br>Members of a community advisory board representing community-based organizations serving persons living with HIV/AIDS<br><br>Exclusion criteria:<br>NR	Agencies are interested in education about linkages between HIV/AIDS and cancer;<br>Cancer care providers should be culturally competent and understand the needs of PLWHA;<br>Agencies see opportunities to improve their services by participating in research but are wary about loss of autonomy and irrelevant research findings.	CAB members drawn from one source (the Collaboration's CAB), comprising only 10 ASOs among the 14 invited;<br>Members may not represent the broader population of ASOs and their staff;<br>Findings may not reflect overall ASO perspectives in New York City, New York State, or the country;<br>Study was a formative/qualitative step, requiring a future quantitative survey for confirmatory data.
3	Inclusion criteria:<br>PLWH: 18 years or older; comorbidity of diabetes and/or hypertension diagnosed after HIV; NCD diagnosis between five years and three months prior to the interview (to reduce recall bias but ensure sufficient experience of receiving NCD care);<br><br>CTC HCPs: directly cared for PLWH; at least six months of work experience;<br>OPD HCPs: directly cared for patients with diabetes or hypertension; at least six months of work experience	Organisational/healthcare system factors comprised the most barriers overall (four out of six sub-themes): lack of access to diagnostic equipment, and lack of continuity of NCD care were barriers for early diagnosis and/or safe effective care. Education on lifestyle behaviours and counselling on ARV adherence were the only facilitators (for prevention). Individual factors comprised four sub-themes, three of which were facilitators: self-monitoring of NCDs by PLWH (for safe effective care). HCPs' knowledge was simultaneously a barrier for prevention; PLWH knowledge was a barrier for prevention and safe	Only PLWH currently receiving HIV care were recruited; PLWH not retained in care may have different or additional barriers to care for diabetes and hypertension<br>Only PLWH with a comorbidity known to the CTC were recruited; many diagnoses of diabetes and hypertension are likely unknown to CTC HCPs, meaning included PLWH may be more comfortable with CTC HCPs or more outspoken about their conditions<br>Only six PLWH were recruited from Makole Health Centre due to difficulties in recruitment (CTC and

	Exclusion criteria:<br>NR	effective care.<br>Syndemic factors comprised three sub-themes, all barriers: Syndemic factors were the most prevalent barriers across all three components of care combined.	OPD HCPs were evenly split across both facilities, but PLWH were not)<br>Study conducted in an urban setting, though both facilities care for PLWH across the predominantly rural Dodoma region.
4	Inclusion criteria:<br>Key informants: facility managers and healthcare providers at the eight selected primary healthcare facilities<br>Participants recruited until data saturation was reached<br>Eight of the 12 primary healthcare facilities in Harare purposely selected (six urban, two rural)<br><br>Exclusion criteria:<br>NR	Urban facilities scored higher than rural facilities across almost all domains;<br>Training readiness: mean 79.2% urban, 75.0% rural;<br>Basic equipment availability: 55.6%–89.9% (rural facilities scored lowest);<br>Laboratory/on-site testing readiness: 40% rural, 56.7% urban;<br>Essential medicines availability: 71% urban, 64% rural;<br>Strong readiness for HIV screening, prevention, ART management and PMTCT (83%–100%);<br>Satisfactory readiness for T2DM care: mean 92% (both urban and rural);<br>Notable discordance in staffing between urban and rural facilities; mean core health staff = 43% of total workers.	Study conducted only in Harare (capital city), limiting generalisability to other regions of Zimbabwe;<br>Focus on urban and peri-urban/rural facilities within one city only;<br>Perspectives of patients with HIV and T2DM were not directly collected (only healthcare providers were interviewed);<br>The study relied on facility audits and key informant interviews, without patient-level data.

5	Inclusion criteria:<br>Age ≥ 18 years;<br>HIV diagnosis at least 1 month previously (confirmed by original or photocopy of laboratory results or physician documentation in the medical record);<br>Luganda or English speaking;<br>Access to a mobile telephone for follow-up interview.<br><br>Exclusion criteria:<br>NR	97% of participants would have preferred to receive HIV care at the cancer center;<br>39% of participants at follow-up reported barriers to care; reporting any barrier was associated with premature withdrawal from cancer treatment (P = .003);<br>25% of follow-up participants had prematurely stopped cancer care;<br>15% missed ART for at least 1 week between cancer diagnosis and initiation of cancer treatment;<br>19% reported HIV providers were unaware of their cancer diagnosis.	No comparator arm of HIV-negative patients (study designed as preparation for implementation studies);<br>Selection bias: only patients aware of their HIV diagnosis and who ultimately initiated cancer care were included; not representative of all persons with HIV-associated malignancy, especially those with undiagnosed HIV or those who never entered cancer care;<br>Majority of patients lived relatively close to Kampala, were receiving ART, were engaged in HIV care, and had access to a cellphone — inherent bias of recruiting from a tertiary referral center;<br>Self-report of HIV care and events before registration at the cancer center, with attendant possibility of social desirability bias;<br>Recall bias: significant time had elapsed between initial presentation and study participation;<br>No robust death registry in Uganda outside of Kampala; ascertainment of date of death was dependent on proxy report or censoring at last care episode.
6	Inclusion criteria:<br>Patients receiving treatment for HIV, hypertension and diabetes in the health facilities 6 months before the initiation of the integrated model in 2011<br>Age ≥ 18 years<br>Seven operational managers who were professional nurses-in-charge of the selected health facilities<br><br>Exclusion criteria:<br>Patients being managed for other chronic diseases	ICDM pilot facilities had a 5.7% greater likelihood of controlling patients' CD4 counts than comparison facilities (coef = 0.057; 95% CI: 0.056, 0.058; P < 0.001);<br>CD4 count control was 0.2% greater in pilot than comparison facilities during 24 months of ICDM implementation (coef = 0.002; P < 0.001);<br>Pilot facilities had a 1.0% greater chance of controlling BP than comparison facilities (coef = 0.010; P = 0.002);	Incomplete or unavailable facility-level data;<br>Paucity of information on facility-level factors such as comparative data on staffing and patient load;<br>Inability to obtain at least eight data time points before the integrated model was commenced;<br>Extrapolations could not be made concerning (dis)satisfaction of professional nurses with services in the integrated model due to small number of facility managers interviewed (seven);<br>Study findings may not reflect PHC facilities in urban

	(not HIV, hypertension or diabetes);<br>Minors less than 18 years of age;<br>The elderly with reduced capacity for comprehension as observed during the informed consent process.	Pilot facilities had a 3.6% greater chance of controlling BP than comparison facilities during 24 months of ICDM implementation (coef = 0.036; $P < 0.001$);<br>BP control remained suboptimal (<50%) in pilot facilities, indicating the purpose of the integrated model had not yet been fully achieved;<br>Mediation pathway best fit the Donabedian SPO model (ranked 1st across goodness-of-fit criteria).	municipalities in Gauteng, Mpumalanga and North West provinces where the pilot model was also being implemented;<br>Study sample in the qualitative research was not randomly selected and may not represent patients in the selected health facilities;<br>The qualitative study did not allow the establishment of cause and effect relationships;<br>Analysis for diabetes patients could not be undertaken due to small sample size (two in each study arm).
7	Inclusion criteria:<br>HCWs employed at the ART clinic of Wentworth Hospital;<br>Directly involved in the management of HIV and NCDs;<br>Eligible HCWs included medical officers, medical interns, and nurses;<br>All HCWs working in the ART clinic were invited to participate; those who consented were enrolled.<br><br>Exclusion criteria:<br>NR	Patients often prioritised HIV care over NCD management, partly because health education strongly emphasised HIV adherence;<br>Patient satisfaction was mixed: "I think our patients are happy and others are not happy. Some of the complaints that you get, you will get a patient who is complaining about the long waiting time or staff attitude" (FGD 5);<br>Integrated care was associated with stigma reduction;<br>The findings informed the implementation of integrated HIV–NCD services at the facility in 2025.	Not all medical officers participated; only those directly involved in HIV and NCD management were included;<br>Time constraints limited participation by some HCWs, potentially narrowing the diversity of perspectives;<br>Findings cannot be generalised to all HCWs in similar settings, as the study focused on one urban district hospital;<br>The data were collected in 2017, which raises concerns about timeliness; however, "the challenges and contextual factors identified remain relevant, as many of the same systemic issues persist".
8	Inclusion criteria:<br>Older persons (age 50 or greater) living with HIV;<br>Living in Langa or Khayelitsha communities, Cape Town;<br>Initiated on ART.<br><br>Exclusion criteria:<br>NR	Patients were generally unaware of options such as linked appointments or Chronic Care Clubs;<br>Access to ART was relatively efficient (through adherence clubs), but NCD chronic care services were associated with long waits and many challenges;<br>Despite policy changes towards integrated care, this was not the experience of OPLWH in these communities;<br>The 'ART advantage' was noted: linkage to HIV care was associated with diagnosis and treatment of NCDs.	Small, non-representative sample;<br>Sampling strategy (referrals from HIV service and convenience sampling at clinics) may have missed individuals with fewer or greater barriers;<br>Those with fewer barriers may have accessed HIV care through the Community Dispensing Unit or Community Health Workers;<br>Those with greater barriers may not have been attending services at all (unknown HIV status, non-adherent, or too unwell to access services);;<br>Respondents may be more likely to be people who prioritise ART care, missing those who prioritise

			NCD care or do not know their HIV status<br>Attempts to include equal numbers of men and women and respondents under and over 60 years of age were challenging;<br>Findings likely underestimate the potential barriers brought on by bifurcated care;<br>Other geographical and provincial settings may present different or additional challenges not considered in this paper.
9	Inclusion criteria:<br>Adult (≥18 years);<br>HIV-positive, recently postpartum (6–18 months prior to enrollment);<br>Previously diagnosed with a NCD that required further evaluation after delivery.<br><br>Exclusion criteria:<br>NR	Some mothers reported being treated with more respect at infant care clinics than at non-infant care clinics;<br>Participants were much more likely to seek advice about the baby's care from the clinic than about HIV or NCD care;<br>Only one of the 25 participants reported talking to clinic staff about HIV or NCD; most turned to family and community;<br>Disclosure to an HIV-seroconcordant partner was associated with increased positive outlook on self-care and clinic visits;<br>Baby care was identified as a potential entry point for improving maternal care after delivery.	Results represent findings from one city in South Africa (not generalizable);<br>One-time interview: "The one-time nature of our interview means that we cannot assess how attitudes, motivations, or actions may change over time";<br>Recall bias: "We asked postpartum women to describe both their recent past care and their current care; past care may have been affected by recall bias".
10	Inclusion criteria:<br>Radiation, medical, or surgical oncologists;<br>Academic oncologists: recently had a consultation with a patient with both HIV and cancer (assessed through real-time medical record review), interviewed within 2 weeks of the consultation;<br>Community oncologists: recruited from an online oncology community if they had a consultation with a patient with both HIV and cancer in the past 2 years (allowed to self-report patient encounters);<br>Written informed consent obtained from all participants;<br>Enrollment continued until thematic saturation was	Most participants stated they viewed HIV like any other chronic condition or comorbidity and did not endorse generalizing treatment recommendations based on HIV status;<br>Collaboration with ID doctors was not always routine; many only reached out when pertinent medical information was not available in the EMR;<br>Some oncologists believed patients living with HIV experience more socioeconomic issues, though many acknowledged this was not the case for all patients;<br>Participants had minimal discussion with patients about the effect of cancer on HIV or vice versa.	Most participants were recruited from 2 large academic medical centers; perspectives may differ in other health systems or settings with fewer resources;<br>Electronic medical record screening algorithm may have excluded patients referred from community clinics;<br>Possibility of recall bias, particularly for community practice oncologists given the time lapse between consultation and interview;<br>Generalizability is limited given the study's small sample size;<br>Social desirability may have influenced participant responses despite assurances of anonymity;

	reached.<br><br>Exclusion criteria:<br>NR		The study only included oncologists and did not capture perspectives of other providers in a patient's clinical care team or patient perspectives.
11	Inclusion criteria:<br>Service users enrolled or receiving care from the HIV, hypertension and diabetes clinics at the participating facilities;<br>Two users with a combination of conditions (HIV with hypertension or diabetes, or hypertension and diabetes) per facility;<br>Three users with a single condition (HIV, diabetes or hypertension) per facility;<br>HCWs (doctors, medical assistants and nurses) engaged in HIV and NCD care delivery at the participating facilities;<br>Two HCWs purposively selected from each site.<br><br>Exclusion criteria:<br>NR	Users' knowledge about their conditions improved with the establishment of the integrated clinic, attributed to intensified joint HIV/NCD patient health education sessions;<br>Most users reported that waiting time greatly reduced after integration; users accessing care through the integrated clinic were often prioritised first at the laboratory and dispensing window;<br>All users with multi-morbidities reported that the integrated clinic reduced their number of visits, saving transport money and time;<br>Most users reported comfort with the sitting arrangements under the integrated clinic;<br>Most users reported improved HCW–patient relationships in the integrated clinic;<br>As MOCCA was coming to an end, some drugs were beginning to run out again;<br>Overall, care users and healthcare providers welcomed the integrated model.	Differences across facilities in terms of the level and type of healthcare services offered and/or integration;<br>MOCCA as a feasibility study provided drugs and diagnostic support covering shortages in NCD drugs: "We therefore do not know how available, affordable and acceptable the integrated HIV/NCD healthcare delivery model would be without this support";<br>Loss to follow-up was anticipated and additional participants were recruited to address this.
12	Inclusion criteria:<br>Aged ≥ 18 years;<br>In HIV care for ≥ 12 months prior to the start of the study;<br>Diagnosed with hypertension and/or diabetes in the parent study;<br>Aware of the HTN/DM diagnosis;<br>Had previously sought care for HTN/DM at any health facility.<br><br>Exclusion criteria:<br>NR	None of the participants received HTN medication at the HIV CTC;<br>HTN/DM care outside the CTCs was perceived as complex, uncoordinated, and inadequately tailored to the needs of people with multimorbidity;<br>Medication cost was the primary reason for treatment discontinuity;<br>Younger participants preferred lifestyle modifications; older participants preferred herbal therapies;<br>Participants expressed strong preference for integrated HIV/NCD care at the HIV CTCs;<br>Coping strategies included: seeking financial support	Recruitment exclusively from six high-volume HIV CTCs in an urban setting, limiting generalizability to other contexts;<br>Participants were already in HIV care, potentially missing perspectives of ALHIV with HTN/DM outside the service;<br>Access and availability of services as reported by participants were not confirmed;<br>Control of participants' comorbid conditions was not assessed<br>HIV CTC providers' perspectives were not included.

		from family, community, and church members; use of nearby community pharmacies; home-based symptom monitoring; use of old prescriptions to purchase medications.	
13	Inclusion criteria:<br>All 25 health centres and five hospitals supported by the EQUIP (Extending Quality Improvement for HIV/AIDS in Malawi) project in two rural northern districts of Malawi;<br>NCD coordinators who had been in their position for at least 12 months (for the qualitative interviews).<br><br>Exclusion criteria:<br>13 health centres not supported by the EQUIP project at the time of the study (eight in one district, five in another).	100% of hospitals and 92% of health centres had uninterrupted supply of hydrochlorothiazide; only 40% of hospitals and no health centres had uninterrupted supply of metformin;<br>Screening for hypertension among ART patients was only conducted at one hospital and no health centres;<br>48% (12/25) of health centres provided ART and NCD treatment in the same consultation;<br>NCD coordinators saw their main role as providing clinical care rather than developing and supporting NCD services at health centres;<br>NCD coordinators strongly supported dedicated NCD clinics as the preferred model; they were hesitant about full integration with ART services, citing stigma and workload concerns;<br>Only two hospitals (40%) and two health centres (8%) submitted quarterly NCD reports to the NCD unit.	In completing the cross-sectional survey, the most senior person on duty that day was interviewed; in some cases, this was not the person in charge of the facility;<br>Health centres were purposefully selected as EQUIP-supported sites; they may be better resourced than other facilities in the district;<br>The two districts have lower population densities and are less urban than other regions in Malawi; findings may not be generalisable to other districts;<br>Only three qualitative interviews were conducted, representing only the views of NCD coordinators; views of clients and communities were not reflected.
14	Inclusion criteria:<br>Age ≥ 18 years;<br>Diagnosed with HIV and on ART;<br>Diagnosed with and receiving treatment for T2DM;<br>Attended PHC facility for > 12 months;<br>Able to provide written informed consent.<br><br>Exclusion criteria:<br>Not receiving active treatment for either HIV or T2DM;<br>Attending the facility for less than 12 months;<br>Unable to provide informed consent (e.g., due to cognitive impairment).	Integrating HIV and T2DM care at all PHC levels;<br>Training providers on managing co-morbidities and patient-centred care;<br>Standardizing appointment systems for multiple conditions;<br>Subsidizing T2DM medication and diagnostics;<br>Implementing eHealth strategies to reduce patient burden.	The study was limited to Harare and may not reflect deep rural experiences;<br>Interviews were self-reported and subject to recall or desirability bias;<br>The small sample size limits generalizability but enables in-depth understanding;<br>The study focused only on HIV and T2DM, excluding other comorbidities such as hypertension.

15	Inclusion criteria:<br>Adult patients (18 years and older);<br>With a previous or recent diagnosis of HIV, diabetes, or hypertension, alone or in combination;<br>Followed up in primary health care units in Tanzania and Uganda.<br><br>Exclusion criteria:<br>NR	The findings suggest that the integrated care model may appeal to older patients, and those with multiple chronic conditions and that the model will achieve near-universal retention in care in these sub-groups.	The study sample may have been biased toward older participants and/or those living with multiple chronic diseases. Additionally, participant follow-up was impacted by disruptions caused by the COVID-19 pandemic. Finally, the study lacked a comparison group.
16	Inclusion criteria:<br>Patients with breast cancer who had completed systemic treatment at Princess Marina Hospital in Gaborone;<br>Participants who had also completed other multimodality treatment, including surgery and/or radiation therapy at the time of enrollment.	"Acceptance and de-stigmatization" were important facilitators reported by participants. Additional facilitators included trust in the care team and social support, which importantly included peer support from other survivors. Other facilitators were adequate knowledge, high self-efficacy and perceived role as a survivor advocate. Finally, in PWH, being able to integrate care or simplify treatment regimens for both cancer and HIV, promoted adherence to cancer treatment.	This study was limited to women with breast cancer which may limit generalizability to other cancer types. However, this distribution of participants is representative of the distribution of PWH and late-stage breast cancer presentation in Botswana and other countries in SSA. Furthermore, breast cancer experience and recommended surgical interventions are unique to these group of patients with cancer and therefore some aspects of social stigma identified in relation to breast cancer surgery and risk of infertility are not generalizable to the experience of other patients with cancer. Finally, stigma is in part driven by social norms which are specific to cultural contexts, and therefore experiences of breast cancer stigma or intersectional stigma in this population may not be globally generalizable.
17	NR	Neither aging nor HIV are always at the forefront of women's concerns when they discuss their illness self-management. Comorbid conditions, specifically diabetes and hypertension, were perceived to be more difficult to self-manage than HIV. This difficulty was not primarily attributed to aging or HIV, but rather to daily life struggles such as reduced income and health insurance and the very nature of paid work.	The participants for this study are all active participants in a longitudinal study regarding HIV among women in the United States. Consequently, the cohort effect may have influenced women's perceptions of how aging is influencing their HIV and co-morbid illness management. Thus, these findings may not be generalizable to all older African American women living with HIV. Furthermore, 9 of the 23 participants reported ever having AIDS. This is important to note because variations in AIDS status could influence women's perceptions and practices

			regarding their HIV and co-morbid illness self-management.
18	Inclusion criteria:<br>Healthcare practitioners who have worked for more than one year with PLHIV and could provide verbal consent were eligible to participate;<br>Patients were eligible to participate in the study if they were 50 years of age or older, living with HIV and at least one NARC, and on ART for a period of no less than 6 months.<br><br>Exclusion criteria:<br>NR	Diabetes Mellitus and hypertension were most frequently recognised in older PLHIV;<br>Patients described NARCs, HIV and ART medication as major risk factors to their health. Ageing, however, was often not considered a risk factor to a person's life.	The study sample was small and selective. In addition, the sampling strategy was bound to the availability of participants in their ART clinic and the time of recruitment. Potential bias may have occurred as respondents were found through the clinics, which could have attracted more motivated and engaged health care providers. Moreover, part of the research team has committed to the English version of the transcript and not the Amharic version, which may not capture sensitive or culturally specific findings. In addition, this diverse sample is limited to Southern Ethiopia which may limit the generalisability of findings to other geographic areas.
19	Inclusion criteria:<br>Aged 18 years or above;<br>Living with an HIV chronic comorbidity;<br>On ART and receiving treatment at the selected health facilities.<br><br>Exclusion criteria:<br>NR	Several challenges continue to exist with delivering the three outcomes of the ICDM model. In this study, these included under-resourced facilities and poor adaption of the guidelines on the delivery of the model;<br>Patient had concerns over non-disclosure, polypharmacy, socio-economic challenges and the lack of support programmes for managing HIV chronic comorbidities that affect the ability of PLWH and other chronic diseases to receive integrated chronic care at the PHC level.	The length of time the ICDM model was implemented in each of the sampled healthcare units in this study was not assessed. Consequently, the suboptimal ICDM model results mentioned in this study may reflect units that have adopted it recently. The conclusions of this study are representative of patients and healthcare professionals in only two selected provinces of South Africa. The results should be interpreted taking into account the limitations of the data collection methods due to restrictions imposed during the COVID-19 pandemic.
20	Inclusion criteria:<br>18 years of age or older;<br>Received care at a participating HIV clinic;<br>Had a recent HIV viral load that was < 200 copies/ml;<br>Had hypertension (systolic blood pressure > 130 mmHg twice in the past 12 months and/or were taking anti-hypertensive medication);<br>Had hypercholesterolemia (defined as a non-HDL cholesterol > 130 mg/dL or on cholesterol-lowering	HIV specialty clinics have adeptly evolved in order to provide primary care and CVD prevention, which is quite different than their original focus on opportunistic infections and AIDS. The primary funding source of these clinics – the Ryan White HIV/AIDS Program – has been slower to evolve proactively to the growing CVD burden;<br>For those HIV providers who do not provide primary care, working closely with the patient’s primary care	Purposive sampling methods were used, which may increase the risk of bias. Data not included in the interview guide emerged inductively during conversations with the interviewer, showing that the ability to describe additional relevant topics was limited. Variability exists among insurance benefit managers, with many PLHIV being users of public insurance, which may limit the transferability of this data.

	medication).<br><br>Exclusion criteria:<br>NR	provider can strengthen their (primary care provider and PLWH) relationship, which may increase the patients' prevention behaviors;<br>It is possible that HIV and primary care teams can work together to develop a new model of value-added care for PLWH that synergistically improves both HIV and cardiovascular outcomes for this vulnerable population.	
21	Inclusion criteria:<br>Adults over 18 years old;<br>Had documented HIV-infection and a diagnosis of either DM or HTN or both;<br>Had attended MACs for HIV care or had been on ART for at least 6 months before they were enrolled into IC;<br>Had been enrolled in ICs for at least 12 months as of August 2017.<br><br>Exclusion criteria:<br>NR	The adherence to group visits (club attendance) was high and sustained at 1-year of attending IC;<br>At 1-year post IC registration, 93.1% of population was retained in care;<br>HIV control was sustained at 1-year post IC enrolment with optimal viral suppression near 100%;<br>NCD care can be safely incorporated into HIV care programs without compromising HIV care;<br>In addition to increased efficiency in terms of optimizing utilization of resources, the integration of HIV and NCD care has also been reported to be convenient and acceptable to patients.	Socioeconomic variables known to independently affect the control of hypertension and diabetes mellitus were not available in the patients' routine data. A single blood pressure measurement, recorded in medical records, was used to assess control 12 months after inclusion in the control group. Almost 7% of patients were lost to follow-up (6.9%) 12 months after inclusion in the control group. An indirect indicator of adherence was used, which may not accurately capture actual adherence and its impact on the control of non-communicable diseases.
22	Inclusion criteria:<br>≥ 18 years;<br>Spoke English or a local African language;<br>Diagnosed with HIV;<br>On ART;<br>Had documented hypertension;<br>Received care at a participating clinic.<br><br>Exclusion criteria:<br>NR	The primary barriers to implementing hypertension screening and treatment identified in this study were the lack of operational capacity and siloed flow of operations in clinics and the limited value placed on hypertension care guidelines;<br>Inadequate training on hypertension care guidelines was frequently cited as a barrier in hypertension care management;<br>Linking patient medical record data to an information dashboard summarizing provider- and clinic-level hypertension care metrics may help motivate clinicians to follow hypertension care guidelines;<br>The ability to practice task-sharing to some degree was identified as a key factor in facilitating the adoption and implementation of hypertension screening and care in primary care clinics.	This study is limited by the fact that interview and discussion guides were not developed to elicit data on each TDF domain, which likely accounts for the absence of data for several TDF domains. Additionally, the COVID-19 pandemic caused interruptions and changes in data collection methods that may have hindered participation and altered participant's perceptions regarding health services.

23	Inclusion criteria:<br>Adult people with HIV (age ≥18 years);<br>On suppressive ART;<br>With high blood pressure with or without hyperlipidemia.<br><br>Exclusion criteria:<br>Taking anti-hypertensive medications solely for a non-hypertension indication such as heart failure;<br>Severe hearing or speech impairment or other disability that would limit participation in the intervention component;<br>Being in a nursing home or receiving inpatient psychiatric care;<br>Terminal illness with life expectancy <4 months;<br>No reliable access to a telephone;<br>Pregnant, breast-feeding, or planning a pregnancy during the study period;<br>Planning to move out of the area in the next 12 months;<br>Non-English speaking.	The number of participants who were at evidence-based blood pressure goals increased by over 50% by study end;	The study lacked a control group in its design. The sample size was relatively small, and approximately one-quarter of the participants did not have data for the full 12 months. The study was not designed to significantly evaluate subgroups within the population, which limited the ability to identify individuals who might respond favorably or unfavorably to the intervention. The population also presented relatively well-controlled blood pressure, as only about one-third of the participants had baseline systolic blood pressure (SBP) above 140 mmHg. Home blood pressure measurements were used, with variability in measurement technique.
